# The residual life of bendiocarb on different substrates under laboratory and field conditions in Benin, Western Africa

**DOI:** 10.1186/1756-0500-6-458

**Published:** 2013-11-13

**Authors:** Armel Djènontin, Odile Aïmihouè, Michel Sèzonlin, Georgia B Damien, Razaki Ossè, Bhonna Soukou, Gil Padonou, Fabrice Chandre, Martin Akogbéto

**Affiliations:** 1 Faculté des Sciences et Techniques/Université d’Abomey Calavi, Cotonou, Bénin; 2 Centre de Recherche Entomologique de Cotonou (CREC), 06 BP 2604 Cotonou, Bénin; 3 Centre de Recherche Entomologique de Cotonou (CREC)/MiVEGEC (IRD 224-CNRS 5290-UM1-UM2), 01 BP4414 RP Cotonou, Bénin; 4 MiVEGEC (IRD 224-CNRS 5290-UM1-UM2)/Laboratoire de lutte contre les Insectes Nuisibles (LIN), 911 Ave Agropolis BP 64501, 34394 Montpellier, Cedex 5, France

**Keywords:** Indoor residual spraying, Bendiocarb, Residual life, Substrates, Laboratory, Field

## Abstract

**Background:**

The efficacy of bendiocarb against pyrethroid resistant *An. gambiae* and the residual life of this insecticide on different substrates were evaluated under laboratory and field conditions.

**Methods:**

Bioassays according to the WHO (World Health Organization) standard protocol were carried out on different substrates impregnated with bendiocarb. Data were analyzed using a binomial regression model with R software.

**Results:**

A good efficacy of the bendiocarb against pyrethroid resistant *An. gambiae* and a high variation of its residual life according to the surfaces treated was observed. The probability that a female mosquito died after exposure to a treated substrate was below 80% after 13 weeks for the teak wood; 7 weeks for the wall made with a mixture of sand and cement and 6 weeks for walls made with red clay and those made with a mixture of the red clay and cement.

**Conclusions:**

Considering the residual life of bendiocarb on walls made with red clay, the main substrates treated during IRS campaigns in rural areas in Benin, more than 2 treatments rounds per year would be necessary to achieve a long term efficacy of IRS using bendiocarb in these areas. Financial and logistical resources required to achieve such levels of coverage need more political will from leaders of African endemic countries. While waiting for innovative malaria control tool, alternative insecticides or combinations of insecticides have to be used for insecticide resistance management in Benin.

## Background

Malaria control represents one of the most public health challenges in Tropical Africa. It causes 655,000 deaths per year and constitutes a strong burden for the development of sub-Saharan countries [1]. Since no effective vaccine is available, the use of Indoor Residual Spraying (IRS) and Long Lasting Insecticide Treated Nets (LLIN) remained the mainstay of malaria prevention [2]. Larval Source Management (LSM) is also an important component of malaria prevention in appropriate settings [3]. Initially, IRS was considered as useful for malaria prevention in areas with low-to-moderate transmission whereas LLINs were considered as suitable in high endemic areas [3]. Only pyrethroids are insecticides used for net impregnation because these products have strong efficacy, fast acting effect at low dose and low toxicity for mammals [4]. Concerning IRS, various insecticides class are used (DDT (dichlorodiphenyltrichloroethane), pyrethroids, organophosphates, carbamates, etc.). Scaling up of LLINs coverage in Africa countries has become the cornerstone of malaria prevention. Unfortunately, pyrethroid resistance in malaria vectors populations has spread across Africa [5] and the World Health Organization (WHO) had now changed its policy, promoting wide application of IRS even in highly endemic areas [3]. Numerous studies have suggested the use of carbamates (or organophosphates) and the combination of both LLINs and IRS (using carbamate) in the same dwelling as alternative for pyrethroid resistance management in malaria vectors [6-9]. A drastic decrease of malaria transmission was even observed on field the months following a large scale IRS using bendiocarb protecting more than 350,000 peoples [10,11]. However, many authors have questioned the global efficacy of IRS in high malaria transmission settings [12]. A recent randomized controlled trial carried out Southern Benin demonstrated that using a combination of LLINs and annual IRS with bendiocarb in the same dwelling showed no additional benefit compared to LLIN alone on malaria transmission and morbidity [13]. The IRS intervention in this study was however done at a small scale (7 hamlets treated protecting about 3,000 inhabitants). To explain these results, one of reasons evoked by authors is the residual life of bendiocarb on treated substrates. Indeed, to be sustainable in malaria-endemic countries, IRS must be re-applied frequently and it is important to know the optimal application interval on field depending on the residual life of the bendiocarb. Previous studies have reported that insecticide residual life of an insecticide depends on the substrate that it is applied to [14,15]. Since various different substrates are treated in a community during IRS implementation campaigns, it would be useful to determine the residual life of insecticide on these substrates.

Here, we present results of a study aiming to determine efficacy of bendiocarb against pyrethroid resistant *An. gambiae* and the residual life of this insecticide on different substrates under laboratory and in field conditions.

## Methods

### Mosquitoes

The susceptible strain “Kisumu” of *An. gambiae* originate from Kenya and bred in the insectary of the Entomological Research Centre of Cotonou (CREC) was used for the bioassays. A local population of *An. gambiae*, considered as a group of pyrethroid-resistant individuals (knock-down resistant (*kdr*) allelic frequency > 80%), collected as larvae and raised to adulthood in the insectary was also used [16]. This population strongly resistant to pyrethroids is susceptible to carbamate [17].

### Insecticides

A wettable powder (WP 80 W FICAM) formulation of bendiocarb was used. This insecticide is an irreversible acetylcholinesterase inhibitor acting on the insect central nervous system [18]. Bendiocarb was provided free of charge by Bayer Environmental Science (Lyon, France).

### Study area and substrates treated

The field study was carried out in Ouidah-Kpomassè–Tori (OKT) health district in southern Benin. Details of this study area were already described [13]. During a randomized controlled trial carried out in twenty eight villages of this health district, IRS using bendiocarb was implemented in seven villages [13]. Before implementation of vector control, a complete household census was done. During this census, all substrates to be treated were identified. Therefore, four different substrates were recorded. These substrates were the following:

1) wall made with the local red clay used for house building in rural areas in southern Benin (RC);

2) wall made with a mixture of cement and the local red clay (CRC);

3) wall made with a mixture of cement and sand (CS);

4) door and window made with a teak wood (TW).

### Treatment of substrates

Under laboratory conditions, substrates observed in the field were imitated. Then, blocks (thick: 2 cm and diameter: 10 cm) were made with of RC, CRC, CS and TW. These substrates were impregnated with bendiocarb at the dosage of 400 mg/m^2^ using a manual sprayer. Sprayed blocks were stored at ambient temperature in the laboratory during testing procedures. On field, substrates were impregnated at the same dosage by the Research Triangle Institute (RTI) team, the implementing partner of the United States Agency for International Development in Benin.

### Bioassays

Under laboratory conditions, bioassays were carried out on blocks impregnated with bendiocarb twice in a month. In the field, tests were carried out monthly on walls (and window or door made with teak wood) of four houses randomly selected in the study area. Untreated surfaces were used as control. The efficacy and the residual life of the bendiocarb on each impregnated substrate was evaluated using WHO cone tests [19]. This test consists of introducing ten to fifteen unfed two to five-day-old female mosquitoes into a plexiglas cone attached to the insecticide-treated material for 30 minutes. After exposure, the mosquitoes were placed in small cups, provided with sugar solution and maintained at 27 ± 2°C with a relative humidity of 80 ± 10% for 24 hours to assess delayed mortality. Tests were considered as invalid and repeated when control mortalities exceeded 20%. When control mortalities were less than 20%, but exceeded 5%, a correction of mortality was made using Abbot’s formula [20].

### Data analysis

A binomial regression model was used to estimate the probability for a female of *An. gambiae* to die when exposed to a treated substrate according to the number of weeks after treatment, taking into account the pyrethroid resistance status of the mosquitoes and the substrate treated. The number of weeks after which this probability would decrease below 80% was estimated with 95% confidence interval. Analysis was done using R software (version 2.11.1).

## Results

In total, 328 bioassays were performed using 4,264 *An. gambiae* females of which 2,145 *An. gambiae* “Kisumu” and 2,119 wild *An. gambiae* (local population). Mosquito mortality rates observed after exposure to the bendiocarb treated substrates according to the number of weeks after treatment are presented in Tables 1 and 2. The first week after treatment, there was 100% mortality recorded among both susceptible and pyrethroid resistant mosquitoes whatever is the treated surface, in both the laboratory and the field. However, in following weeks, the number of dead mosquitoes after exposure to treated surfaces varied considerably indicating a decrease in the availability of the bendiocarb on these treated surfaces.

**Table 1:** Mosquitoes mortality after exposition to bendiocarb treated substrates under laboratory according the time post-treatment

				***Anopheles gambiae *** **“Kisumu”**								**Local population of *Anopheles gambiae***							
**N weeks after treatment**				**1**	**3**	**5**	**7**	**10**	**13**	**15**	**18**	**1**	**3**	**5**	**7**	**10**	**13**	**15**	**18**
**Substrates and treatments**	**Red clay**	Control	N tested	12	10	11	10	10	15	15	13	10	15	12	11	12	14	12	10
			% mortality	8	0	0	0	0	0	0	0	0	0	0	0	0	0	0	0
		Treated	N tested	14	10	11	12	15	10	14	14	13	14	20	12	15	14	14	19
			% mortality	**100**	**100**	**100**	**83**	**13**	**10**	**7.1**	**7.1**	**100**	**100**	**100**	**8.3**	**33**	**21**	**7.1**	**5.3**
	**Cement + red clay**	Control	N tested	11	10	10	15	15	10	10	15	10	14	12	13	12	11	12	10
			% mortality	0	0	0	6	0	0	0	0	0	0	8	0	0	0	0	0
		Treated	N tested	14	12	14	9	14	15	18	15	12	12	10	11	11	12	18	15
			% mortality	**100**	**92**	**100**	**33**	**21**	**20**	**17**	**13**	**100**	**100**	**100**	**36**	**9.1**	**8.3**	**5.6**	**6.7**
	**Cement + sand**	Control	N tested	14	15	11	15	16	10	10	11	12	14	12	10	12	11	10	10
			% mortality	7	0	0	0	0	0	0	0	0	7	0	0	0	0	0	0
		Treated	N tested	12	10	10	12	22	14	20	15	14	15	15	12	14	14	11	17
			% mortality	**100**	**100**	**100**	**50**	**45.5**	**42.9**	**40**	**13.3**	**100**	**100**	**100**	**75**	**71.4**	**42.9**	**18.2**	**11.8**
	**Teak wood**	Control	N tested	10	10	14	15	11	15	15	13	11	12	11	12	10	12	14	12
			% mortality	0	0	0	0	0	0	0	0	0	0	0	8	0	0	0	0
		Treated	N tested	10	10	10	15	14	12	18	17	12	12	13	12	16	12	15	14
			% mortality	**100**	**100**	**100**	**100**	**100**	**100**	**83.3**	**29.4**	**100**	**100**	**100**	**100**	**100**	**100**	**46.7**	**21.4**

**Table 2:** Mosquitoes mortality after exposition to bendiocarb treated substrates in the field according the time post-treatment

				***Anopheles gambiae *** **“Kisumu”**					**Local population of *Anopheles gambiae***				
**N weeks after treatment**				**1**	**5**	**9**	**13**	**17**	**1**	**5**	**9**	**13**	**17**
**Substrates and treatments**	**Red clay**	Control	N tested	14	12	10	10	11	10	14	12	10	13
			% mortality	0	0	0	0	0	0	0	0	0	0
		Treated	N tested	98	48	55	57	52	56	53	55	63	60
			% mortality	**100**	**37.5**	**5.45**	**5.26**	**1.92**	**100**	**7.55**	**27.3**	**3.17**	**3.33**
	**Cement + red clay**	Control	N tested	14	12	13	10	11	15	12	14	15	10
			% mortality	7	0	0	0	0	0	0	0	0	0
		Treated	N tested	60	36	45	54	55	57	47	29	54	48
			% mortality	**100**	**22.2**	**2.22**	**3.7**	**1.82**	**100**	**10.6**	**13.8**	**1.85**	**0**
	**Cement + sand**	Control	N tested	10	15	12	10	13	10	10	12	14	15
			% mortality	0	7	0	0	0	10	0	0	0	0
		Treated	N tested	53	48	46	49	45	44	39	47	63	48
			% mortality	**100**	**100**	**30.4**	**26.5**	**17.8**	**100**	**100**	**70.2**	**23.8**	**18.8**
	**Teak wood**	Control	N tested	12	15	15	14	15	14	12	14	12	10
			% mortality	0	7	0	0	0	0	8	0	0	0
		Treated	N tested	40	57	52	60	58	59	60	54	55	65
			% mortality	**100**	**100**	**100**	**83.3**	**46.6**	**100**	**100**	**100**	**70.9**	**35.4**

**Figure 1:**
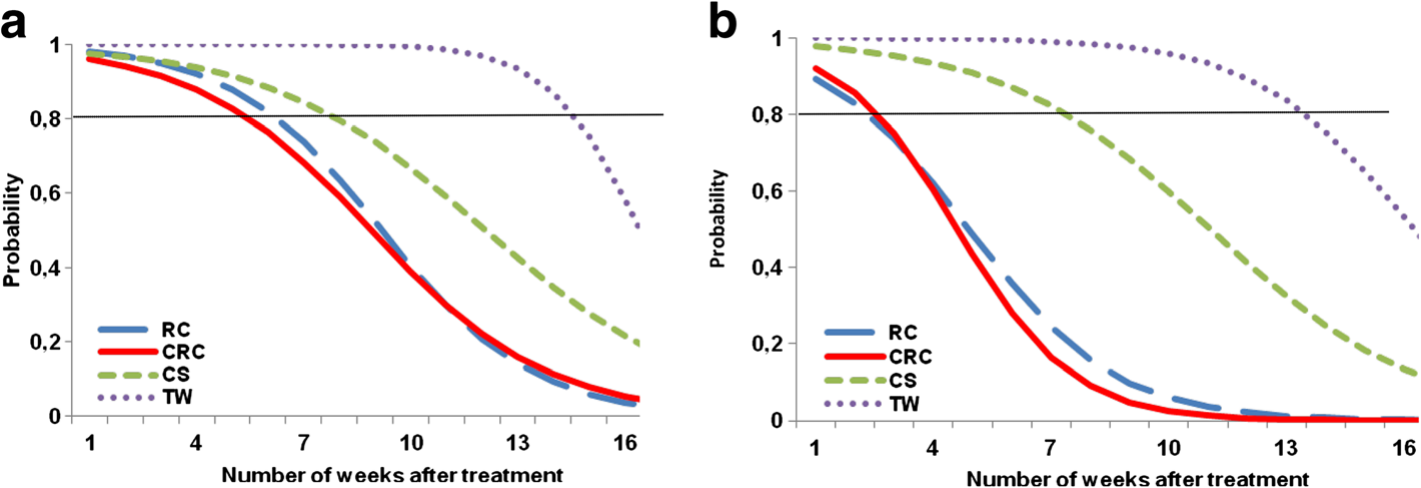
**Probability of mortality among *An. gambiae *** **after exposure to different bendiocarb treated subtrates under laboratory (a) and field (b) conditions using WHO cone assays, according the number of weeks after treatment.**

The binomial regression model showed that the efficacy of bendiocarb on the local population of *An. gambiae* resistant to pyrethroid does not significantly differ from the susceptible strain of this mosquito (OR = 0.742 [0.495-1.106], p = 0.145). Under laboratory and field conditions, the residual life of bendiocarb significantly differed according to the substrate that was treated (p < 0.001). Using this model, the residual life of the bendiocarb on the different treated substrates was estimated in terms of the probability of mortality among female mosquitoes exposed to these substrates as a function of weeks after treatment (Figure 1). In the laboratory, the probability that a female mosquito that was exposed to a treated substrate died was below 80% after 15 [14-16] weeks for the teak wood; 8 [7-9] weeks for the wall made with a mixture of sand and cement and 6 [5-7] weeks for walls made with red clay and those made with a mixture of the red clay and cement. In the field, residual life observed was lower. Respectively 13 [12-14] weeks and 7 [6-8] weeks after treatment, the probability of mortality among female mosquitoes exposed to the teak wood and to wall made with a mixture of sand and cement impregnated with bendiocarb was below 80%. Regarding the walls made with red clay and those made with a mixture of the red clay and cement, this number did not differ and was 6 [5-7] weeks.

## Discussion

The present study has assessed the efficacy of bendiocarb against pyrethroid resistant *An. gambiae* and the residual life of this insecticide on different substrates under laboratory and field conditions. The bendiocarb had a good efficacy against pyrethroid resistant *An. gambiae.* However, this efficacy decreased rapidly through the time and a high variation of the residual activity was observed according to the surfaces treated.

The efficacy of the bendiocarb against pyrethroid resistant *An. gambiae* is interesting since pyrethroid resistance via metabolic mechanisms (e.g. monooxygenases), as showed on *Anopheles funestus*, could lead to a cross resistance between carbamates and pyrethroids [21]. Results obtained in the present study could mean that target site pyrethroid resistance mechanism plays an important role in pyrethroid resistance of malaria vectors tested. Indeed, pyrethroids target channel Na + voltage-dependent (NaVdp) whereas carbamates act on synapse acetylcholinesterase (AChE) [22]. Nevertheless, these results have to be interpreted cautiously and do not probably mean that carbamate resistance mechanisms are not present in wild population of pyrethroid resistant *An. gambiae* tested in the present study. Indeed, genetic diversity that characterizes species is a key factor for interpretation of such results. It constitutes a major genetic basis of resistance to insecticides in vectors populations. The natural populations of mosquitoes could include a few rare resistant individuals which are difficult to identify because of their low number within the populations. The use of insecticides disrupting the natural environment could lead to ecological changes. In such environments, genetic traits previously giving a competitive advantage may no longer be advantageous in the new conditions. Then, an artificial selection follows the introduction of insecticides. Consequently, a new genetic and ecological structuring occurs in the populations to allow individuals to be adapted henceforth to the new environmental conditions. Insecticides will then be the favorable factors to the artificial selection resistance allele [23,24]. Moreover, *Ace*^*1R*^ mutation (conferring to mosquitoes resistance to carbamate) is recorded in malaria vectors populations in southern and northern Benin [25,26]. The current level of the frequency of *Ace*^*1R*^ mutation (~ 10%) does not probably represent a veritable threat for vector control using carbamate. However, the presence of this mutation in *An. gambiae* populations is worrying since its spread would represent a veritable threat for vectors control using carbamate and then for the management of pyrethroid resistance in malaria vectors. Susceptibility to carbamate in the field population of malaria vectors is a valuable natural resource and has to be preserved through suitable pyrethroid resistance management strategies such as rotation, mixture and mosaic of insecticides with unrelated mode of action. Then, monitoring of *Ace*^*1R*^ mutation in malaria vectors and researches on others mechanisms involved in resistance to carbamate in Benin are desirable.

The variation of the residual life of the bendiocarb according to the surfaces treated observed in the present study confirms previous observations [14,15]. The residual life of bendiocarb on walls made with red clay and mixture of red clay and cement was lower. Indeed, the porosity of these substrates is greater than that of the other substrates. The efficacy of bendiocarb decreases below 80% on walls made with red clay 5 weeks after treatment. This residual life of bendiocarb is lower than that observed on mud in others studies. Bioassay carried out by Ansari *et al*. (2004) in India revealed a persistence of bendiocarb against *Anopheles culicifacies* at 100% mortality for about 10 weeks on mud whereas Mpofu *et al*. (1991) in Zimbabwe showed that bendiocarb provided 74% mosquitoes mortality up to 5 months after spray on mud [14,27]. In Cameroon, Etang and colleagues reported that 13 weeks after spray on mud, the estimated efficacy of bendiocarb in terms of *An. gambiae* s.s. killing was 80% [15].

To plan an effective indoor residual spray program for Benin, it is essential to know the residual life of the insecticide used. Since more than 80% of houses in rural areas in this country are made with red clay [13], and that the efficacy of bendiocarb decreases below 80% on walls made with red clay 6 weeks after treatment, a minimum of 2 treatments rounds per year suitably scheduled would be necessary to achieve a long term efficacy of IRS using bendiocarb in rural areas in Benin. Financial and logistical resources required to achieve such levels of coverage need more political will from leaders of African endemic countries. Moreover, bendiocarb is an expensive insecticide [28] that adds significantly to the cost of such intervention. It is important to indicate that as the resistance to pyrethroids has spread in malaria vectors, resistance to carbamates will also have spread if these insecticides are used repeatedly in the same area.

## Conclusions

The bendiocarb is effective against pyrethroid resistant *An. gambiae* tested in the present study. The residual life of this insecticide on walls made with red clay, the main substrate treated during IRS campaigns in Africa rural areas, is however too low. Consequently, more than 2 treatments rounds per year would be necessary to achieve a long term efficacy of IRS using bendiocarb in these areas. While waiting for innovative malaria control tool, alternative insecticides or combinations of insecticide have to be used to delay bendiocarb resistance selection.

## Abbreviations

WHO: World Health Organization; IRS: Indoor residual spraying; LLIN: Long lasting insecticide treated nets; LSM: Larval source management; DDT: Dichlorodiphenyltrichloroethane; CREC: Entomological research centre of Cotonou; Kdr: Knock-down resistant; WP: Wettable powder; OKT: Ouidah-Kpomassè–Tori health district; RC: Wall made with the local red clay; CRC: Wall made with a mixture of cement and the local red clay; CS: Wall made with a mixture of cement and sand; TW: Door and window made with a teak wood; RTI: Research triangle institute; NaVdp: Channel Na + voltage-dependent; AChE: Acetylcholinesterase; Ace1R: Acetylcholinesterase resistant mutation; PMI: President’s malaria initiative.

## Competing interests

The authors declare that they have no competing interests.

## Authors’ contributions

AD designed the study, participated in data analysis, discussed the results and drafted the manuscript. OA carried out bioassays and contributed to the manuscript draft. MS contributed to results discussion and helped to draft the manuscript. GD contributed to results discussion and helped to draft the manuscript. RO contributed to data collection and helped to draft the manuscript. BS carried out statistical analysis and helped to draft the manuscript. GP contributed to results discussion and helped to draft the manuscript. FC contributed to the study design and results discussion. MA contributed to the study design, supervised data collection, contributed to results discussion and helped to draft the manuscript. All authors read and approved the final manuscript.

## Authors’ information

**AD**, PhD, Medical Entomologist

**OA**, MSc, Medical Entomologist

**MS**, PhD, Population Geneticist

**GD**, MD, PhD student, Epidemiologist

**RO**, PhD, Medical Entomologist

**BS**, MSc, Statistician

**GP**, PhD, Medical Entomologist

**FC**, PhD, Medical Entomologist

**MA**, Pr, Medical Entomologist
